# How Does Subjective Social Status Associate With Depression Among the Labor Force Population in China? — Analysis of the Mediation Effect Based on the Sense of Social Equity

**DOI:** 10.3389/ijph.2025.1607942

**Published:** 2025-10-17

**Authors:** Hong Zhang, Xiaohui Ren, Yongzhao Zhou

**Affiliations:** ^1^ Integrated Care Management Center, West China Hospital, Sichuan University, Chengdu, Sichuan, China; ^2^ Department of Health Behavior and Social Medicine, West China School of Public Health and West China Fourth Hospital, Sichuan University, Chengdu, China

**Keywords:** labor force population, depression, subjective social status, mediation, sense of social equity

## Abstract

**Objectives:**

With the rising prevalence of depression and its growing disease burden, and given that few studies have examined the link between subjective social status (SSS) and depression among the labor force, this study aimed to explore the association between SSS and depression in the workforce and to examine potential mediating factors.

**Methods:**

We analyzed data from the 2018 China Labor-force Dynamic Survey, with a final sample of 10,065 participants. Depression was assessed using the 20-item Center for Epidemiologic Studies Depression (CES-D) scale. SSS was measured via the MacArthur Scale. Linear regression models examined the association between SSS and depression, while structural equation modeling tested the mediating effect of sense of social equity.

**Results:**

In total, 12.37% of participants were identified as having probable depression. SSS was significantly associated with depression (*β* = −0.08, *P* < 0.05). Sense of social equity accounted for approximately 33% of the total effect.

**Conclusion:**

Among China’s labor force population, SSS is independently related to depression, and sense of social equity plays an important mediating role. Depression in the labor force - those with low SSS - warrants greater attention.

## Introduction

Depressive disorder, also known as depression, is clinically characterized by a persistently low mood or a marked loss of interest in daily activities [1]. It represents a significant global health burden with complex clinical presentations and far-reaching consequences. Extensive research has established a robust association between depressive symptoms and an increased risk of suicidal ideation [2], with longitudinal studies demonstrating that individuals exhibiting higher baseline depression levels are more likely to engage in subsequent self-harm behaviors [3]. Depression frequently coexists with significant somatic disturbances, particularly sleep dysregulation. Clinical evidence suggests that sleep problems and disorders are more prevalent among depressed patients, and may not only exacerbate existing symptoms but also contribute to treatment resistance and higher relapse rates [4]. Characteristic symptoms often accompany depressive episodes, with the loss of appetite induced by melancholia following a specific pattern [5] that frequently results in clinically significant weight loss [6]. Additionally, depression also exerts substantial influence on physical health outcomes, with meta-analytic data indicating a 23%–83% increased mortality risk among cancer patients (including those with lung, breast, colorectal, and prostate malignancies) [7]. This elevated risk may be partially explained by the multidimensional effects of depression, including amplified pain perception [8], reduced treatment adherence [9], and dysregulation of neuroendocrine-immune pathways [10]. Epidemiological data reveal the staggering global impact of depressive disorders. The World Health Organization (WHO) estimated that in 2015, depression affected 322 million individuals worldwide, representing 4.4% of the global population [11]. Regional studies have revealed substantial variations in the prevalence rates of depression. For instance, the China Mental Health Survey, which included 32,552 participants in 201, reported a lifetime prevalence of depressive disorders of 6.8% [12]. In the United States, a 2019 depression screening found that approximately 7.8% of American adults (equivalent to 19.4 million individuals) had experienced depression [13]. Meanwhile, the prevalence of depression across European countries ranges from 5% to 10% [14]. Against this backdrop, projections suggest that major depressive disorder will become the primary contributor to the global disease burden by 2030 [15].

Depression is a common mental disorder with varying prevalence rates among different demographic groups. WHO data show higher rates among women and older adults [11]. Vulnerable groups include adolescents [16], postpartum women [17], students [18], older adults [19], and chronically ill patients [20]. With an estimated 3.63 billion workers worldwide (45% of the global population) [21], the prevalence of depression varies significantly by occupation, highlighting the need for targeted workplace interventions [22].

A growing body of research has established subjective social status (SSS) - defined as an individual’s self-perceived position in the socioeconomic hierarchy [23, 24]- as an important psychosocial determinant of depression [25–28]. However, emerging evidence suggests that this relationship varies across populations and cultural contexts. Among university students in Ghana, higher SSS was associated with significantly lower depression levels, with analysis revealing important interactive effects between students’ disposable income and age in moderating this association [27]. Similarly, a UK population study demonstrated that SSS predicted depression risk among adults aged 52 years or older, with this association remaining significant after controlling for gender and objective socioeconomic indicators [25]. Valuing autonomy was hypothesized to moderate the relationship between SSS and depression, as evidenced by U.S. research showing that the SSS-depression association is attenuated among individuals who strongly value personal autonomy [26].

In the United States, the prevalence of depression and anxiety among women was found to be approximately twice as high as among men [29]. Previous research has documented gender differences in the association between SSS and depression. For instance, in South Africa, SSS explained 82% of the variance in female depression, a proportion significantly higher than the 44% observed in men [30]. Similarly, among U.S. adults aged 33–44, SSS exerted a stronger inhibitory effect on depression in women compared to men [31]. However, some studies have found that the association between SSS and depression was significant in both men and women, with no evidence indicating gender differences in the strength of this association [25]. Additionally, a longitudinal study revealed that SSS at age 15 was associated with depression at ages 18, 21, and 28, and that the gender difference in the association between SSS and depression tends to disappear with increasing age. Specifically, gender differences emerged at ages 18 and 21 - with a stronger association observed in girls - whereas by age 28, the relationship between SSS and depression showed no significant gender variation [32]. It can be seen from these results that existing studies have not yet reached a consistent conclusion on whether there is a gender difference in the association between SSS and depression.

Sense of social equity reflects a citizen’s evaluation of and attitude toward the current state of social justice, which is shaped by both objective social structures and subjective psychological experiences [33]. Social psychological theories posit that SSS emerges through continuous social comparison processes [34]. Individuals with lower SSS often perceive restricted access to educational, economic, and social resources compared to their peers, which can potentially foster feelings of inequity and related psychological consequences [35]. Notably, empirical evidence confirms that sense of social equity significantly influences wellbeing outcomes and demonstrates positive associations with life satisfaction among Chinese primary and secondary educators [36]. These established relationships raise a crucial research question: Could sense of social equity mediate the association between SSS and depression? Utilizing nationally representative data from the China Labor-force Dynamics Survey, this study examines three research questions: (i) the independent association between SSS and depression in China’s working-age population; (ii) whether there is a gender difference in this relationship; and (iii) the potential mediating role of sense of social equity in the relationship between SSS and depression.

## Methods

### Data Source

The China Labor-force Dynamics Survey (CLDS) is a nationally representative longitudinal study conducted biennially by the Social Science Survey Center at Sun Yat-sen University. Initiated in 2012, the survey employs a multi-stage, stratified probability sampling method to track labor force dynamics in 29 provincial - level administrative regions in China [37]. The 2018 wave (the fourth and most recent available dataset at the time of analysis) collected comprehensive data on working-age populations, including measures of education, employment status, occupational mobility, workplace protections, and subjective wellbeing.

**FIGURE 1:**
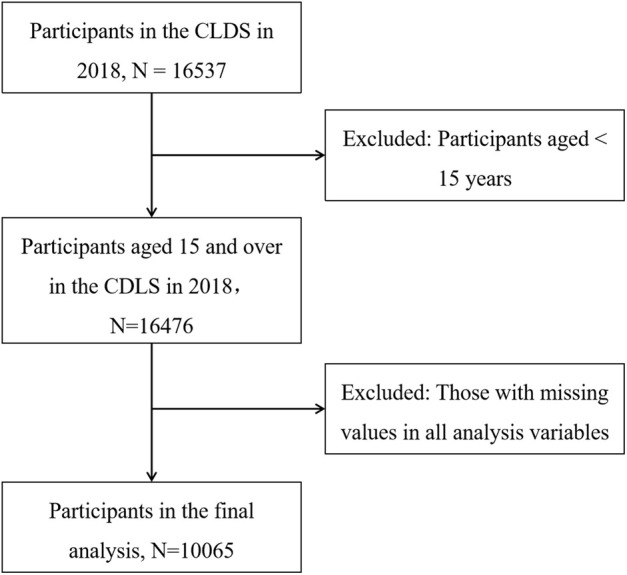
Flowchart showing the selection of the participants in this study (China, 2025).

From the original 2018 CLDS cohort of 16,537 respondents, we applied the following inclusion criteria: (i) aged 15–64, or 65 and older but still employed; (ii) with complete data for all study variables. After applying these criteria and conducting data quality checks, our analytical sample comprised 10,065 participants. The data screening process is shown in Figure 1.

### Depression

Depression was assessed using the 20-item Center for Epidemiological Studies Depression Scale (CES-D), a validated self-report instrument designed to measure depressive symptomatology in the general population [38]. Respondents rated the frequency with which they experienced symptoms during the preceding week on a 4-point scale ranging from 0 (rarely or none of the time) to 3 (most or all of the time), yielding a total score range of 0–60, with higher scores indicating greater symptom severity. Based on a meta-analysis by Gemma Vilagut et al. [39], a cut-off score of 20 was deemed more appropriate for identifying cases of “possible depression or depressed”. Consistent with these recommendations, we established a cut-off score of 20 to optimize case identification. This threshold yielded two categories: no depression symptoms (CES-D ≤ 19) and possible depression or depressed (CES-D ≥ 20). The continuous depression score served as our primary dependent variable in regression analyses.

### Subjective Social Status

The independent variable, subjective social status, was assessed using the validated MacArthur Scale of Subjective Social Status [40]. This instrument employs a graphical representation of a 10-rung ladder, where respondents are instructed: “Imagine this ladder represents where people stand in society. At the top (rung 10) are those who are best off. At the bottom (rung 1) are those who are worst off. Please indicate where you would place yourself on this ladder.” In accordance with the CLDS protocol, responses were coded as a continuous variable ranging from 1 (lowest status) to 10 (highest status). The MacArthur Scale of SSS demonstrated strong psychometric properties in a study of 300 middle-aged and older married U.S. couples, with clear evidence of convergent and discriminant validity [41]. Moreover, the scale has now been widely applied in research involving the Chinese population [42, 43].

### Sense of Social Equity

As a mediator variable, sense of social equity was assessed using a single-item: “How do you think of the current overall social equity?”. Responses were recorded on a 5-point scale ranging from 1 (totally unfair) to 5 (totally fair).

### Covariates

Existing research has demonstrated that occupation is closely associated with SSS in British civil servants and American urban populations. These studies also revealed a stronger association between SSS, education, and income [44]. Based on this evidence, our analysis adjusted for several key covariates, including employment category, annual personal income, education level, self-rated health, and time spent on housework per day. Additional demographic covariates included age, gender, marital status, and urban/rural residence.

Marital status, education level, residence, employment category, annual personal income, and self-rated health were all based on a questionnaire. Marital status was categorized into six groups: single, first-married, remarried, divorced, widowed, and cohabiting. Education level was classified across six levels, ranging from illiteracy to bachelor’s degree or above. Residence was classified as either urban or rural. The employment category was divided into six groups: “Production personnel in agriculture, forestry, animal husbandry, fishing and water resources,” “Heads of party organs, state organs, mass organizations and social organizations, enterprises and institutions,” “Professionals and technical personnel,” “Clerical and related personnel,” Social production service and life service personnel,” and “Manufacturing machine-related personnel.” Annual personal income was divided into five groups: less than 10,000 Yuan, 10,000–30,000 Yuan, 30,000–50,000 Yuan, 50,000–100,000 Yuan, and 100,000 Yuan or more. Self-rated health was assessed through the question: “How would you describe your current health status?”, with five response options from 1 (excellent) to 5 (poor). The age classification was based on the research of Lin Z et al. [45], and was divided into three groups: 15–47 years old, 48–63 years old, and ≥64 years old. Housework time per day was divided into four groups: less than 1 h, 1–2 h, 2–3 h, and 3 h or more. Detailed codes for all categorical variables are provided in Supplementary File 1.

### Statistical Analysis

We employed a comprehensive statistical approach to examine the relationship between SSS, sense of social equity, and depression. Continuous variables were summarized using means and standard deviations, while categorical variables were presented as frequencies and percentages. Prior to analysis, we conducted normality tests on all continuous variables, applying appropriate transformations when necessary to meet normality assumptions. The core analysis utilized a linear regression model to assess the association between SSS and depression. To investigate potential gender differences in this relationship, we included an interaction term between gender and SSS in the model. All regression analyses were performed using Stata, version 15.0. For the mediation analysis, we employed a Structural Equation Model (SEM) with a bootstrap method using 1000 resamples [46] to test whether sense of social equity mediated the relationship between SSS and depression. The Structural Equation Model was conducted using Mplus Version 7.0. All models were adjusted for relevant covariates, and all statistical tests were two-sided. For all effect estimates, either α = 0.05 or 95% confidence intervals are reported.

## Results

### Basic Characteristics of Participants

The analytic sample comprised 10,065 participants. Raw depression scores were skewed, with a median of 4 (interquartile range: 0–12); 12.37% of participants were classified as belonging to the “possible depression or depressed” category. Because depression followed a normal distribution after logarithmic transformation, our study used the logarithmically transformed depression as the dependent variable for analysis. The basic characteristics of the participants are presented in Table 1. The majority of participants were between 15 and 63 years old. Over half were men, and the majority were married (5 out of 6). Nearly three-quarters lived in rural areas, and more than one-third had a middle school education. Social production service and life service personnel formed the largest occupational group. The majority of participants had an annual personal income of 100,000 Yuan or less, and nearly five-sixths participants rated their health as good or better. In total, 40% of the participants spent 2 h or more on housework every day. One-way ANOVA indicated that depression varied significantly across groups defined by age, gender, marital status, residence, education, employment category, annual personal income, self-rated health, and housework time per day.

**TABLE 1:** Basic characteristics of participants and results of correlation analysis (China, 2025).

Variables, n (%)	Value	Depression, (M ± SD)	F
Age			6.75**
15–47 years	4,759 (47.28)	1.50 ± 1.16	
48–63 years	4,197 (41.70)	1.59 ± 1.18	
≥64 years	1,109 (11.02)	1.57 ± 1.19	
Gender			97.25***
Male	5,447 (54.12)	1.44 ± 1.16	
Female	4,618 (45.88)	1.67 ± 1.17	
Marital status			6.04***
Single	823 (8.18)	1.55 ± 1.20	
First-married	8,559 (85.04)	1.53 ± 1.17	
Remarried	218 (2.17)	1.82 ± 1.12	
Divorced	167 (1.66)	1.70 ± 1.26	
Widowed	201 (2.00)	1.81 ± 1.23	
Cohabiting	97 (0.96)	1.31 ± 0.98	
Residence			24.50***
Urban	2,870 (28.51)	1.45 ± 1.18	
Rural	7,195 (71.49)	1.58 ± 1.17	
Education			28.87***
Illiterate	1,064 (10.57)	1.81 ± 1.21	
Primary school	2,645 (26.28)	1.67 ± 1.18	
Middle school	3,427 (34.05)	1.49 ± 1.15	
High school	1,090 (10.83)	1.40 ± 1.15	
Junior college	1,069 (10.62)	1.40 ± 1.15	
Bachelor’s degree or higher	770 (7.65)	1.37 ± 1.16	
Employment category			30.85***
Production personnel in agriculture, forestry, animal husbandry, fishing and water resources	195 (1.94)	1.10 ± 1.10	
Heads of party organs, state organs, mass organizations and social organizations, enterprises and institutions	752 (7.47)	1.40 ± 1.15	
Professionals and technical personnel	327 (3.25)	1.26 ± 1.16	
Clerical and related personnel	2,886 (28.67)	1.46 ± 1.17	
Social production service and life service personnel	4,525 (44.96)	1.69 ± 1.17	
Manufacturing machine related personnel	1,380 (13.71)	1.46 ± 1.16	
Annual personal income			48.22***
Less than ¥10,000	2,429 (24.13)	1.80 ± 1.16	
¥10,000 - ¥30,000	2,785 (27.67)	1.55 ± 1.18	
¥30,000 - ¥50,000	2078 (20.65)	1.46 ± 1.16	
¥50,000 - ¥100,000	2041 (20.28)	1.40 ± 1.16	
¥100,000 or above	732 (7.27)	1.31 ± 1.13	
Self-rated health			305.61***
Excellent	1,839 (18.27)	1.07 ± 1.33	
Very good	4,363 (43.35)	1.37 ± 1.14	
Good	2,512 (24.96)	1.80 ± 1.09	
Fair	1,213 (12.05)	2.25 ± 1.04	
Poor	138 (1.37)	2.71 ± 0.99	
Time spent on housework per day			34.66***
Less than 1 h	3,678 (36.54)	1.43 ± 1.17	
1–2 h	2,277 (22.62)	1.49 ± 1.17	
2–3 h	1,994 (19.81)	1.60 ± 1.17	
3 h or more	2,116 (21.02)	1.74 ± 1.16	

*p < 0.05; **p < 0.01; ***p < 0.001 *¥*: Chinese Yuan. “Depression” denotes the logarithmically transformed depression.

### Association Between Subjective Social Status, Sense of Social Equity and Depression

The mean SSS score was 4.47 ± 1.73, and the average sense of social equity score was 3.28 ± 0.88. Pearson correlation analysis revealed significant correlations between depression and both SSS (*r* = −0.19, *P* < 0.05) and sense of social equity (*r* = −0.22, *P* < 0.05).

### The Results of the Linear Regression Model and Interaction Analysis

**FIGURE 2:**
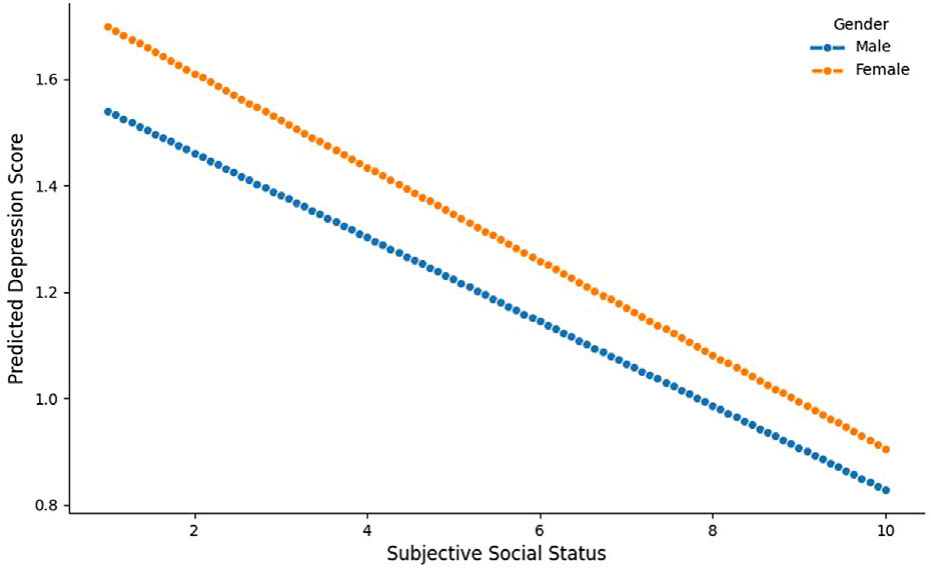
Interaction between subjective social status and gender (China, 2025).

As shown in Table 2, Model 1 examined the association between SSS and depression, with all covariates controlled for. Model 1 indicated a significant correlation between SSS and depression (*β* = −0.08, *P* < 0.05). Building on Model 1, Model 2 further explored the interaction between SSS and gender. It revealed no significant interaction between subjective social status and gender (*β* = −0.01, *P >* 0.05), with the findings visualized in Figure 2.

**TABLE 2:** The results of the linear regression model and the analysis of the interaction between subjective social status and gender (China, 2025).

Variables (reference)	Model 1	Model 2
	*β* (S.E.)	*β *(S.E.)
Subjective social status	−0.08 (0.01)***	−0.08 (0.01)***
Gender* Subjective social status		−0.01 (0.01)
Gender (Male)	0.13 (0.03)***	0.17 (0.06)**
Marital status (Single)		
First-married	−0.12 (0.04)**	−0.12 (0.04)**
Remarried	0.04 (0.09)	0.04 (0.09)
Divorced	−0.03 (0.09)	−0.03 (0.09)
Widowed	0.04 (0.09)	0.03 (0.09)
Cohabiting	−0.32 (0.12)**	−0.32 (0.12)**
Age (15–47 years)		
48–63 years	−0.08 (0.03)**	−0.08 (0.03)**
≥64 years	−0.25 (0.04)***	−0.25 (0.04)***
Residence (Rural)		
Urban	0.07 (0.03)*	0.07 (0.03)*
Education (Illiteracy)		
primary school	−0.04 (0.04)	−0.04 (0.04)
Middle school	−0.11 (0.04)**	−0.11 (0.04)*
High school	−0.13 (0.05)*	−0.12 (0.05)*
Junior college	−0.13 (0.06)*	−0.13 (0.06)*
Bachelor’s degree or higher	−0.06 (0.07)	−0.06 (0.07)
Employment category (production personnel in agriculture, forestry, animal husbandry, fishing and water resources)		
Heads of party organs, state organs, mass organizations and social organizations, enterprises and institutions	0.18 (0.09)*	0.18 (0.09)*
professionals and technical personnel	0.04 (0.10)	0.04 (0.10)
Clerical and related personnel	0.17 (0.08)*	0.17 (0.08)*
Social production service and life service personnel	0.25 (0.08)**	0.25 (0.08)**
Manufacturing machine related personnel	0.17 (0.09)*	0.18 (0.09)*
Annual personal income (Less than ¥10,000)		
¥10,000 - ¥30,000	−0.08 (0.03)*	−0.08 (0.03)*
¥30,000 - ¥50,000	−0.08 (0.04)*	−0.08 (0.04)*
¥50,000 - ¥100,000	−0.11 (0.04)**	−0.11 (0.04)**
¥100,000 or above	−0.08 (0.05)	−0.08 (0.05)
Self-rated health (Excellent)		
Very good	0.28 (0.03)***	0.28 (0.03)***
Good	0.66 (0.03)***	0.66 (0.03)***
Fair	1.05 (0.04)***	1.05 (0.04)***
poor	1.47 (0.10)***	1.47 (0.10)***
Time spent on housework per day (Less than 1 h)		
1–2 h	−0.00 (0.03)	−0.00 (0.03)
2–3 h	0.02 (0.03)	0.02 (0.03)
3 h or more	0.06 (0.03)	0.06 (0.03)

*p < 0.05; **p < 0.01; ***p < 0.001 *¥*: Chinese Yuan.

### Mediation Analysis

**FIGURE 3:**
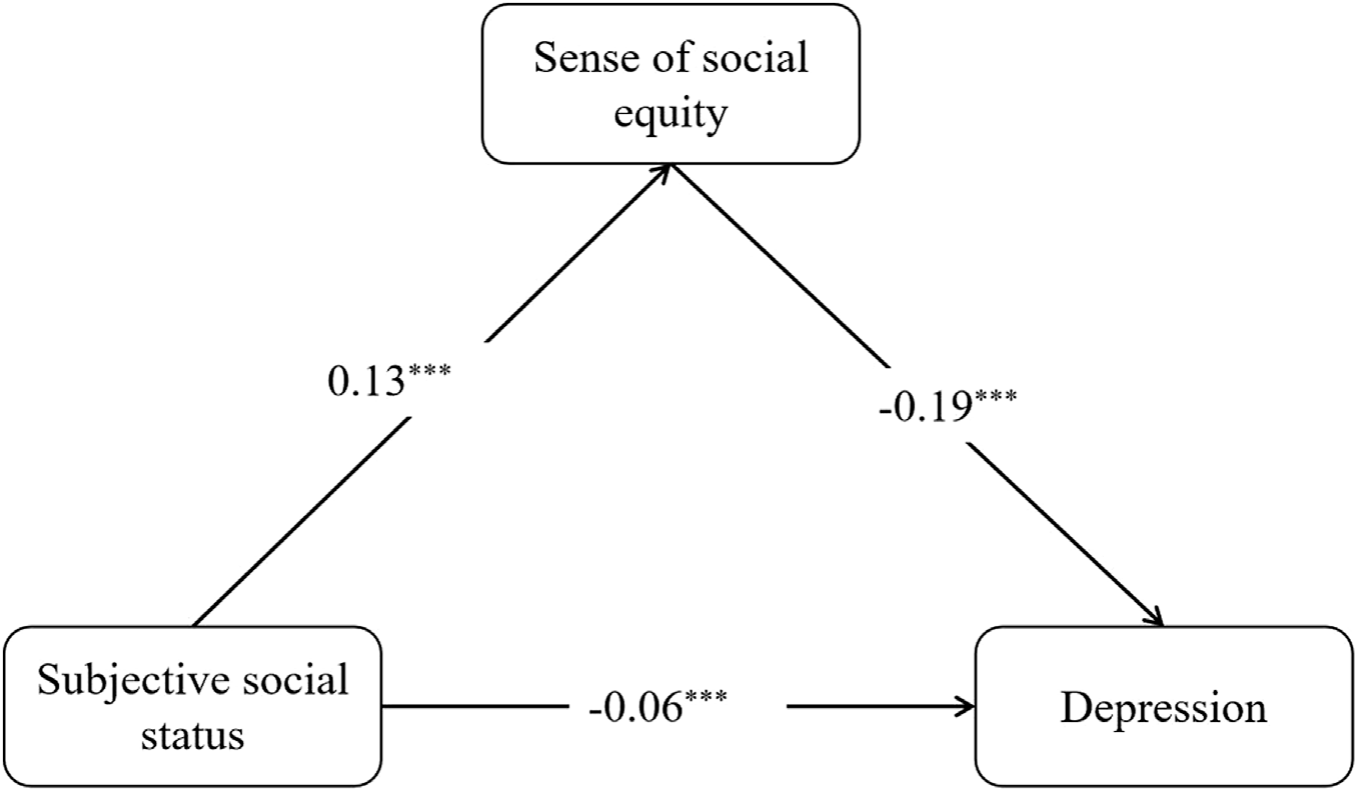
Results of the mediation model (China, 2025). Model fit indices: χ^2^/df = 27.18 (p < 0.05), RMSEA = 0.05, CFI = 0.91, and SRMR = 0.02 *Notes*: *p < 0.05; **p < 0.01; ***p < 0.001.

With all covariates controlled, we examined the mediating role of sense of social equity in the association between SSS and depression using SEM. The SEM analysis revealed significant associations between SSS, sense of social equity and depression. First, we observed that higher SSS was directly associated with both a greater sense of social equity (β = 0.13, *95% CI*: 0.12 ∼ 0.14) and lower depression (β = −0.06, *95% CI*: -0.08 ∼ −0.05) (Figure 3). This indicates that a one-unit increase in subjective social status corresponded to a 0.13-standard-deviation increase in sense of social equity and corresponded to a 0.06-standard-deviation decrease in depression score. Meanwhile, the mediation analysis demonstrated that the sense of social equity was also directly and negatively associated with depression (β = − 0.19, *95% CI*: -0.22 ∼ −0.16). Second, we found a statistically significant indirect effect of SSS on depression through sense of social equity (β = − 0.03, *95% CI*: −0.03 ∼ −0.02), which accounted for approximately 33% of the total effect (β = −0.09, *95% CI*: −0.10 ∼ −0.07). These results suggest that sense of social equity partially mediates the relationship between SSS and depression.

## Discussion

This study provides robust evidence that SSS is significantly associated with depression among China’s working-age population, with sense of social equity serving as a partial mediator in this relationship. Three key findings emerge from our analysis of the 2018 CLDS data: first, we observed a graded inverse association between SSS and depression, consistent with previous studies [25]. Second, approximately one-third of this association was mediated through sense of social equity, suggesting that status-related disparities in mental health may operate through psychosocial mechanisms involving judgments of fairness. Third, these patterns remained consistent across genders.

Our study identified 12.37% of participants as having probable depression, which is lower than the 19.3%–25.5% prevalence reported by Yang et al [47], among Chinese formal and informal employees in 2018. This discrepancy likely reflects methodological differences in depression assessment - while we employed the full 20-item CES-D scale, Yang et al. utilized an 8-item short form, which may capture different severity thresholds. Nevertheless, both studies highlight depression as a significant public health concern among China’s workforce. Notably, our finding that SSS significantly predicts depression risk aligns with present studies in the United States [26] and England involving participants aged 35–86 and 52 years or older [25], respectively.

Our findings align with existing theoretical frameworks that explain how SSS influences depression through both neurobiological and psychosocial pathways. Neurobiological research suggests that low SSS triggers negative emotional responses through limbic system activation, increasing vulnerability to stress-related disorders such as depression [48, 49]. Social psychological theories highlight the role of social comparison - particularly in workplace settings - where individuals frequently and often subconsciously evaluate their status relative to others. These comparisons can lead to perceptions of injustice, occupational inequity, and income disparity, fostering psychological distress [35, 50]. Our study builds on this framework by identifying sense of social equity as a key mediator in the SSS - depression relationship. Individuals with lower SSS tend to perceive greater social inequity, which contributes to life dissatisfaction and ultimately increases depression risk [51]. This mechanism is particularly salient in labor - force populations, where workplace comparisons of status, income, and opportunity reinforce perceptions of unfairness. Together, these findings integrate to demonstrate how SSS disparities translate into mental health outcomes through neurobiological and psychosocial pathways.

Our results revealed a consistent association between SSS and depression across genders, with no significant difference in this relationship. This gender-invariant pattern aligns with the findings of Demakakos P et al [25] and suggests that SSS may function as a universal psychosocial determinant of mental health through similar psychological mechanisms regardless of gender. However, the observed consistency may also reflect a China-specific contextual factor: both men and women in China’s workforce are evaluated by largely gender-neutral occupational status standards, face comparable work pressures and social expectations, and experience similar psychological consequences of economic inequity [52].

### Limitations and Future Directions

This article has several limitations: first, as a cross-sectional study, it only identifies the associations between SSS, sense of social equity and depression, without verifying causal relationships. Second, SSS and sense of social equity were measured via a single item, which may not capture their multi-dimensional nature. Future research should adopt multi-dimensional measures and longitudinal data to explore causality. Third, due to data constraints, workplace factors such as discrimination and regional economic disparities were not included. Future studies should address these to better inform employee mental health interventions. Fourth, 6,472 observations were excluded due to age criteria or missing key variables. A comparative analysis revealed no significant differences in SSS or depression between the included and excluded samples. However, demographic characteristics such as age, marital status, and education differed significantly, which may still limit the generalizability of the findings.

### Conclusion

This study elucidates the significant association between SSS and depression among working-age populations, while identifying sense of social equity as a critical mediating mechanism. Theoretically, our findings establish SSS as an independent psychosocial predictor of depression in the labor-force. Considering the sense of social equity as a mediator clarifies the mechanism linking SSS to depression, enriching our grasp of psychosocial pathways. Practically, our findings underscore that interventions targeting SSS and sense of social equity could mitigate depression across genders, offering a gender-neutral approach to mental health support in the workforce.

First, social comparison environments - particularly workplaces–should be optimized by reducing status-anxiety triggers; this can be done by avoiding public hierarchical evaluations and narrowing visible gaps in income or opportunities. This reduces psychological stress from “relative disadvantages”. Second, sense of social equity should be strengthened. Improving workplace fairness (e.g., transparent promotion systems, reduced discrimination) helps those with low SSS feel less injustice, easing life dissatisfaction and psychological distress, and breaking the “low SSS → perceived injustice → depression” chain.
