# Supplementary material for: How Does Subjective Social Status Associate With Depression Among the Labor Force Population in China? — Analysis of the Mediation Effect Based on the Sense of Social Equity
Source: Int J Public Health. 2025 Oct 17;70:1607942. doi: 10.3389/ijph.2025.1607942 (PMC12575211; doi:10.3389/ijph.2025.1607942)
Supplement: Supplementary file 1 [file Supplementaryfile1.docx]

**Supplementary file 1 variable list and coding**

| **Variable** | **Coding** |
| --- | --- |
| Age | 1 = 15-47 years  2 = 48-63 years  3 = ≥64 years |
| Gender | 1 = Male  2 = Female |
| Marital status | 1 = Single  2 = First-married  3 = Remarried  4 = Divorced  5 = Widowed  6 = Cohabit |
| Residence | 1 = Urban  2 = Rural |
| Education | 1 = Illiteracy  2 = Primary school  3 = Middle school  4 = High school  5 = Junior collage  6 = Bachelor or above |
| Employment category | 1 = Production personnel in agriculture, forestry, animal husbandry, fishing and water resources  2 = Heads of Party organs, state organs, mass organizations and social organizations, enterprises and institutions  3 = Professionals & technical  4 = Clerical and related personnel  5 = Social production service and life service personnel  6 = Manufacturing machine related personnel |
| Annual personal income | 1 = Less than ￥10,000  2 = ￥10,000 - ￥30,000  3 = ￥30,000 - ￥50,000  4 = ￥50,000 - ￥100,000  5 = ￥100,000 or above |
| Self-rated health | 1 = Excellent  2 = Very good  3 = Good  4 = Fair  5 = Poor |
| Time spent on housework per day | 1 = Less than 1 h  2 = 1 - 2 h  3 = 2 - 3 h  4 = 3 h or more |
| Sense of Social Equity | 1 = Totally unfair  2 = Comparative unfairness  3 = Not fair but not unfair  4 = Comparative fairness  5 = Totally fair |
